# Human‐Carnivore Conflicts and Associated Economic Losses in Villages Adjacent to Jorgo‐Wato Protected Forest, Western Ethiopia

**DOI:** 10.1002/ece3.74156

**Published:** 2026-08-09

**Authors:** Mosissa Geleta Erena, Diriba Diba

**Affiliations:** ^1^ Department of Biology Wollega University Nekemte Ethiopia; ^2^ Department of Animal Sciences Wollega University Nekemte Ethiopia

**Keywords:** economic loss, human‐carnivore conflict, livestock depredation, local community, mitigation measures, protected area, protected forest

## Abstract

Human‐wildlife conflict frequently occurs at the borders of protected areas, where the demands of humans and wild mammals greatly overlap. The Jorgo‐Wato Protected Forest is a protected area located in Ethiopia where human‐wildlife conflict is becoming a serious issue for local people. Conflict between humans and carnivores was assessed in the nearby settlements of the Jorgo‐Wato Protected Forest, western Ethiopia. This study aimed to identify carnivore species responsible for livestock depredation, assess the associated economic losses, and examine local strategies used to mitigate human–carnivore conflict around the Jorgo‐Wato Protected Forest. A multistage sampling method was used to evaluate human‐carnivore conflict cases and associated economic costs within the study area. Five villages were purposively selected within a 5 km range of the forest. Data were collected through a household questionnaire survey, key informant interviews, group discussions, and field investigations from November 2019 to September 2021 during both wet and dry seasons. Our results showed that out of the six carnivore species involved in livestock depredation, spotted hyena ( *Crocuta crocuta* ), olive baboon ( *Papio anubis* ), and leopard ( *Panthera pardus* ) accounted for 55.62% (115,576 US$), 20.43% (42,460 US$), and 18.50% (38,454 US$) of livestock and economic loss, respectively, in the study area. To alleviate human‐carnivore conflicts, the local communities utilized guarding (96.25%), chasing with dogs (83.34%), and cleaning shrubs around the home (74.53%) as the common mitigation measures. To sustainably mitigate human–carnivore conflict around the Jorgo‐Wato Protected Forest, coordinated, site‐specific land‐use planning is recommended. This should include developing and strictly implementing a participatory land‐use planning system that clearly delineates wildlife habitat, grazing areas, and settlement zones to minimize overlap between people and carnivores. In addition, buffer zones around the Jorgo‐Wato Protected Forest should be established and expanded to reduce direct encounters between carnivores and livestock.

## Graphical abstract

Conflicts between humans and carnivores were assessed in the nearby settlements of the Jorgo‐Wato Protected Forest. This aimed to identify carnivorous species responsible for livestock depredations, quantify the economic losses resulting from depredations, and identify strategies used by nearby communities to prevent human‐carnivore conflicts in the area.

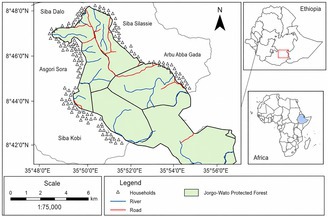

## 1 Introduction

Human–wildlife conflict (HWC) is a common and rapidly growing conservation challenge across the globe (Ladan 2014; Merkebu and Yazezew 2021). It is described as an interaction between humans and wildlife that has an adverse effect on human, social, economic, and cultural well‐being (Bhatta and Joshi 2020; Moni Pradhan and Choudhury 2024). Human–wildlife conflict arises from increasing competition for space and resources as humans and wildlife increasingly share the same landscapes (Nyhus 2016). Globally, HWC can take many different forms, such as attacks by carnivores on people or livestock, crop raiding, livestock‐wildlife disease exchange, carcass poisoning, and retaliatory killing of wildlife (S. Bhatta 2003; Banikoi et al. 2017). Though HWC is a global issue, the intensity differs in location, patterns of land use, and the habits of animal species involved in conflict (WWF 2007). For instance, human population growth, habitat fragmentation and agricultural expansion are key drivers that increase the frequency of human–wildlife conflict (Hill et al. 2002). Habitat fragmentation forces wildlife to move into human settlements, thereby increasing the likelihood of conflict. Climate change further exacerbates this problem by altering rainfall patterns and increasing the frequency of droughts, which disrupt ecosystem productivity and influence wildlife movement (Woodroffe et al. 2005).

In the developing world, such as Africa, HWC have more detrimental effects because people rely heavily on subsistence agriculture and livestock production (Acharya et al. 2016). This makes crops and livestock more vulnerable to wildlife in Africa (Barua et al. 2013). In Africa, livestock vulnerability is intensified by the presence of large and wide‐ranging mammals, which frequently move beyond protected areas in search of food and water (Lindsey et al. 2017). The movement of wild animals out of protected areas forces them to coexist and share resources with humans around these areas (Gusset et al. 2009; Parker et al. 2014). The spatial overlap between humans and wildlife escalates human‐wildlife conflict around protected areas. Moreover, institutional and governance limitations, weak or lack of compensation system, and inadequate land‐use planning often hamper effective conflict mitigation strategies in many African countries (Dickman 2008). In addition, the attitude of human toward wildlife is linked to both economic losses and sociocultural insights, which would influence degree of tolerance and willingness to coexist (Nyhus 2016). As a result, HWC in Africa is characterized by ecological pressures and deep socioeconomic inequalities. This has made it one of the most persistent obstacles to attaining sustainable conservation and human–wildlife coexistence in Africa (Lindsey et al. 2017; Barua et al. 2013).

In Ethiopia, HWC is a significant and growing challenge, especially near protected areas and forest ecosystems. The status of HWC is extensive and ecologically diverse, occurring across highland, midland, and lowland agroecological zones (Yimam 2021; Mamo and Bekele 2022). Human‐wildlife conflicts are a complex, widespread, and increasingly severe socioecological problem that reflects the growing interface between human livelihoods and wildlife habitats. Ethiopia's heavy reliance on agriculture makes livelihoods highly sensitive to wildlife‐related losses (Yazezew 2022). In Ethiopia, crop damage by wild mammals, along with livestock predation, is the most common forms of conflict in rural areas (Biset et al. 2019). The intensity of HWC is increasing, driven by rapid population growth, agricultural expansion, habitat loss, deforestation, and climate variability (Yimam 2021; Yazezew 2022). Evidence from different regions of Ethiopia shows conflicts are frequent and recurrent, undermining household resilience and developing negative attitudes toward wildlife conservation (Yihune et al. 2009). Beyond economic impacts, HWC also affects human well‐being, safety, and social stability, while simultaneously threatening biodiversity through retaliatory killings and habitat degradation (Mamo and Bekele 2022). In Ethiopia, HWC is high in magnitude, increasing in frequency and severity, and deeply linked to broader socioeconomic and environmental changes. Hence, it is requiring integrated, community‐based, and policy‐driven mitigation plans to ensure both sustainable livelihoods and effective wildlife conservation (Yimam 2021).

The issue of HWC has been studied in many protected areas of Ethiopia (Atickem et al. 2010; Demeke and Afework 2013; Yilmato and Takele 2019; Temesgen et al. 2022). However, many protected areas lack adequate empirical studies on HWC. Jorgo‐Wato Protected Forest is one of the most promising wildlife habitats where information about HWC is lacking. Human–wildlife conflicts around the Jorgo‐Wato Protected Forest are likely driven by ongoing human activities including encroachment, shifting cultivation, and livestock grazing within the forest. Similarly, conflicts between humans and carnivores have been escalating, resulting in significant economic losses for communities surrounding the JWPF. Unlike previous studies conducted within established conservation zones, this study examines conflicts in inaccessible landscapes where ecological and socioeconomic data are scarce. Documenting the causes and community perceptions of human–carnivore conflict in such a poorly studied area could fill critical knowledge gaps in these regions. Therefore, the objective of this research was to investigate human‐carnivore conflict and the extent of economic losses to humans from livestock losses caused by carnivores in the adjacent villages of the Jorgo Wato Protected Forest, Western Ethiopia.

## 2 Materials and Methods

### 2.1 Descriptions of the Study Area

**FIGURE 1:**
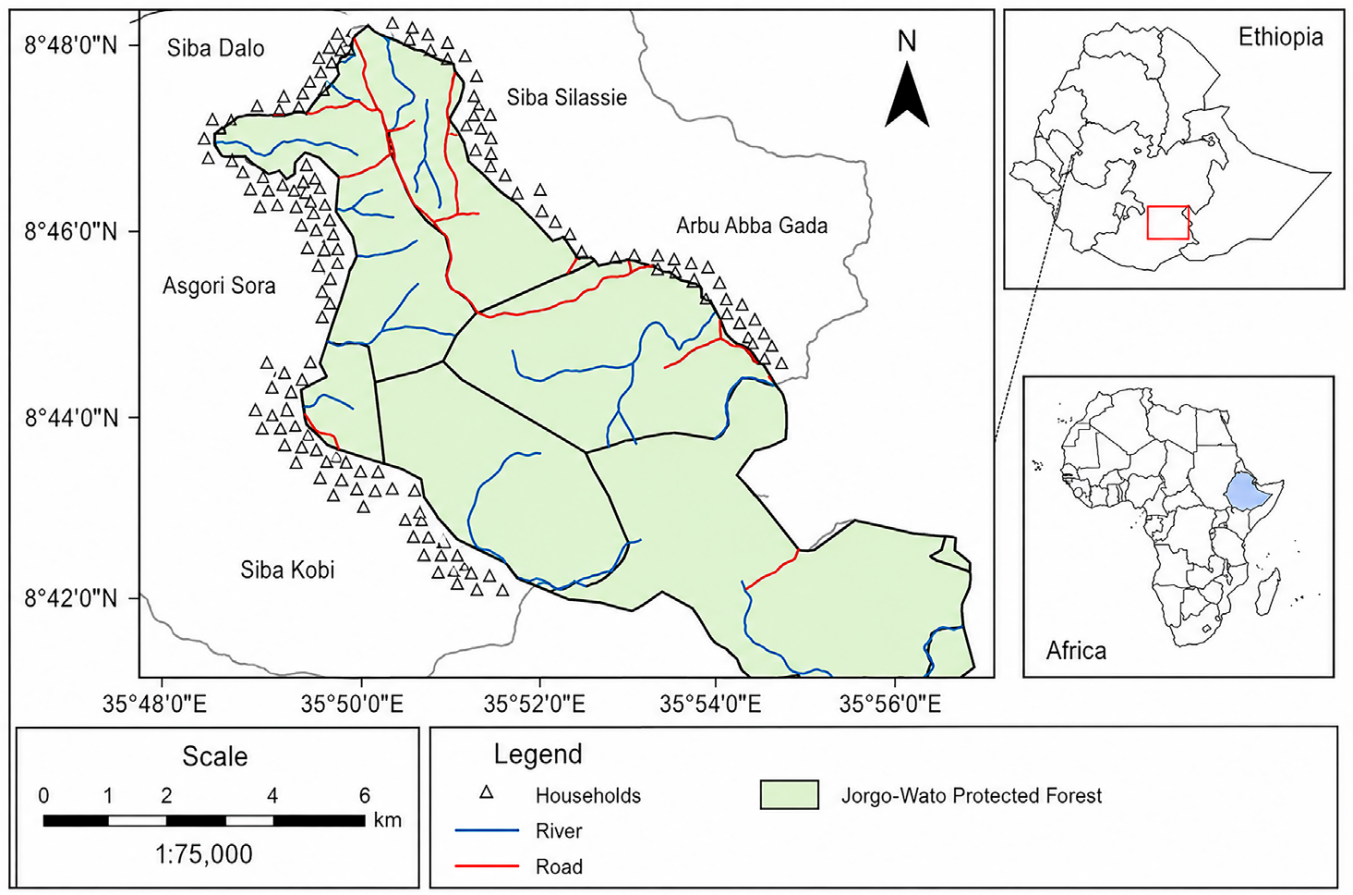
Map of the study area.

The Jorgo‐Wato Protected Forest (JWPF) is situated in western Ethiopia between the Buno Bedelle and west Wollega Zones (Figure 1). The JWPF is located around 509 km west of Addis Ababa, between 8°40′20′′–8°48′06′′ N and 35°48′01′′–35°56′40′′ E. Six farmer associations (lowest administrative structure), which include Arbu Abba Gada, Siba Silassie, Siba Dalo, Asgori Sora, Siba Dalo, and Wato Golbe, surrounded the JWPF (Erena 2022). The elevation of the study area ranges from 1780 to 2584 m asl (above sea level). The region has a bimodal rainfall pattern, with a low rainy season from March to April and a long rainy season from June to September. The shorter dry season lasts from November to February. In the region, the maximum average monthly rainfall was 324 mm in July, and the yearly average rainfall was 1805 mm. February had the highest recorded mean monthly temperature at 28°C, while July and August had the lowest mean temperature at 12°C. The JWPF is home to more than 23 species of mammals, including large mammals such as the African buffalo ( *Syncerus caffer* ), bushbuck ( *Tragelaphus scriptus* ), giant forest hog ( *Hylochoerus meinertzhageni* ), warthog ( *Phacochoerus africanus* ), leopard ( *Panthera pardus* ), spotted hyena ( *Crocuta crocuta* ), common jackal ( *Canis aureus* ), African civet ( *Civettictis civetta* ), caracal ( *Felis caracal* ), African wild cat (*Felis sylvestris*), serval cat ( *Leptailurus serval* ), and common dwarf mongooses ( *Helogale parvula* ).

### 2.2 Methods

#### 2.2.1 Sampling Design

We selected five farmer associations (Arbu Abba Gada, Siba Silassie, Siba Dalo, Asgori Sora, and Siba Kobi) directly bordering JWPF to investigate human‐carnivore conflict, assuming they have more detailed knowledge of human‐wildlife conflicts than those located more than 5 km from the forest. The selection of farmer associations was done through purposive sampling techniques, considering several factors related to HWC. These factors include accessibility for data collection and the documented frequency and occurrence of human–wildlife conflict near the JWPF. The farmer associations that met these criteria were likely more prone to human–wildlife interactions within the context of the human–wildlife conflict, and selected for the study. The villages within each farmer association were also considered for screening based on their proximity to the forest boundary. Distances of villages from the forest were categorized as far (≥ 5 km), medium (3‐4 km), and near (1‐2 km). In each village, all households within 5 km of the forest were selected for the study. Hence, a total of 192 households participated in the study, comprising 45 from Arbu Abba Gada, 47 from Siba Silassie, 49 from Siba Dalo, 27 from Asgori Sora, and 24 from Siba Dalo.

#### 2.2.2 Data Collection

Data were collected from November 2019 to September 2021. Before data collection, we obtained approval from Wollega University and then from the local administrative offices to conduct the study. We clarified that the study primarily aimed to collect factual and pertinent data on conflicts between humans and carnivores in the vicinity of JWPF. Structured and semistructured interviews, focus group discussions, and direct observation were used to collect data from local communities on human‐carnivore conflict around JWPF, following Anderson and Pariela (2005). Prior to data collection, informed consent was obtained from all respondents before conducting the household interviews. Quantitative data were collected from each respondent through house‐to‐house visits using a structured and semi‐structured questionnaire. In addition, key informant interviews (KIIs) and focus group discussions were conducted to gather qualitative data. Key informants were purposively selected for their knowledge, experience, and engagement with human‐carnivore conflict issues in the study area. They included those who lived longer in the area, individuals who owned livestock that had clashed with carnivores before, senior local administrators of farmer associations, and agricultural staff. Focus Group Discussions (FGDs) were conducted in purposive village communities within each farmer association located within 5 km of the JWPF boundary. Participants for the FGDs were selected using a stratified purposive sampling technique to represent various strata of the population affected by human‐carnivore conflict, including male, female, youth, and senior livestock herders. A total of 6–10 participants were used for FGDs.

Given the escalating problem of carnivore attacks, our data collection predominantly focused on conflicts arising from livestock predation by carnivorous animals. Accordingly, a questionnaire was developed to systematically assess the underlying causes, economic implications, and locally adopted mitigation strategies of HWC within the study area. Focus group discussions were conducted in the study area to complement the data collected through household survey. Focus group discussions were conducted in the study area to complement the data collected through the household survey. A total of five focus group discussions were held, one from each selected farmers' association. This approach enabled the collection of detailed qualitative information from each respondent through the use of unstructured questionnaires and open discussions. Participants were purposefully selected based on their extensive experience and more than 10 years of knowledge regarding HWC in the area. All focus group discussions and interviews were carried out using their mother tongue, *Afan Oromo*. In addition to household surveys, direct field observation was employed as a complementary method to document human–carnivore conflict and associated economic losses. Direct observations were carried out within or near the chosen villages as well as the edges of the surrounding forest of the JWPF. These included searching for physical evidence of carnivore occurrence, as well as conflict, carcass remains, carnivore tracks and signs, damaged livestock, and areas of attack. Data were collected during the wet and dry seasons to test seasonal variation in livestock losses.

#### 2.2.3 Data Analysis

Data were analyzed using Origin Version 6 computer software, and Microsoft Excel. The statistical difference in means of variables, such as the total number of cattle lost to different animal species and the financial costs related to such losses, was tested using ANOVA (Zar 2010). We compared the results using the nonparametric Kruskal–Wallis test for economic cost due to different livestock losses to farmers in the study area. All statistical tests were conducted at the significance level of 0.05. A Chi‐square test was applied to assess whether livestock losses were independent of season. The views of home respondents were supplemented and validated by the data gathered from key informant interviews and focus group discussions. Economic losses due to livestock depredation were determined by multiplying the number of livestock lost by the average market price of each species obtained from Bube town, the capital of Nole Kabba District. Accordingly, the average costs of big and medium livestock in the local market were the following: cattle ( *Bos taurus* ; $172), goats ( *Capra aegagrus* ; $90), sheep ( *Ovis aries* ; $110), donkeys ( *Equus asinus* ; $130), horses ( *Equus caballus* ; $200), mules (*Equus mulus*; $240), and chickens (*Gallus domesticus*; $10). The economic loss of livestock attributed to each predator was also estimated. The monetary losses are presented in US dollars, where the exchange rate of 1$ = 50 Ethiopian birr at that time.

## 3 Results

### 3.1 Socioeconomic Characteristics of Respondents

We interviewed 192 people. Most respondents (76.56%) were male; 73.95% were married; and 48.44% were illiterate. Ninety‐three (48.44%) had attended less than grade 6; 34 (17.71%) had attended grades 7–10; and 18 (9.38%) had attended grades 10–12. The majority of respondents (81.25%) reported that raising both crops and livestock was a key source of income, whereas 64.58% and 56.25% relied mainly on crop production and livestock rearing, respectively. The distance between human settlements and their farmlands and the forest was categorized as near (1‐2 km), medium (3‐4 km), or far (≥ 5 km). Accordingly, about 68 (35.42%) of all respondents were near the forest, 95 (49.48%) were at a medium distance, and 29 (15.10%) were far from the forest. All respondents whose farmlands were categorized as near reported livestock depredation. However, about 84.21% and 51.72% of respondents categorized as medium and far, respectively, reported HWC in the study area. Livestock depredation (49.48%) and crop damage (33.33%) were the major types of HWC around JWPF (Appendix S1).

### 3.2 Livestock‐Depredating Carnivores

Eleven carnivorous species and raptors were reported as livestock predators or crop raiders in the study area. These include olive baboons ( *Papio anubis* ), common jackals ( *Canis aureus* ), leopards ( *Panthera pardus* ), grivet monkeys (*Cercopithecus aethiops*), spotted hyenas ( *Crocuta crocuta* ), wildcats (*Felis sylvestris*), African buffalo ( *Syncerus caffer* ), crested porcupines ( *Hystrix cristata* ), blue monkeys ( *Cercopithecus mitis* ), bush pigs ( *Potamochoerus larvatus* ), common duikers ( *Sylvicapra grimmia* ), and raptors such as steppe eagles ( *Aquila nipalensis* ), tawny eagles ( *Aquila rapax* ), and black eagles (*Aquila verauxii*). According to respondents, spotted hyenas (98%), olive baboons (87%), and leopards (71%) were the most problematic predators, responsible for significant livestock losses in the study area (Table 1). Moreover, *Papio anubis* (99%), *Potamochoerus larvatus* (96%), and *Chlorocebus aethiops* (83%) were identified as the most common crop raiders around the JWPF. The olive baboon was particularly notable, reported as both a major livestock predator (87%) and the most frequent crop raider (99%) in the study area.

**TABLE 1:** Lists of problematic animals reported by the local respondents around JWPF.

Predators			Crops raiders		
Common Name	Scientific name	%	Common name	Scientific name	%
Hyena	*Crocuta crocuta*	98	Olive baboon	*Papio anubis*	99
Olive baboon	*Papio anubis*	87	Bushpig	*Potamochoerus larvatus*	96
Leopard	*Panthera pardus*	71	Grivet monkey	*Chlorocebus aethiops*	83
African wildcat	*Felis sylvestris*	56	African buffalo	*Syncerus caffer*	69
Common jackal	*Canis aureus*	32	Porcupine	*Histrix cristata*	55
Raptors Steppe eagle Tawny eagle Black eagle	*Aquila nipalensis* , *Aquila rapax* *Aquila verauxii*	30	Common duiker	*Sylvicapra grimmia*	28
			Blue monkey	*Cercopithecus mitis*	13

Spotted hyenas caused the most cattle losses (*n* = 298). Olive baboons primarily preyed on sheep (*n* = 257), followed by poultry (*n* = 159) and goats (*n* = 140). Leopards predominantly attacked small livestock, particularly sheep (*n* = 167) and goats (*n* = 156), while raptors frequently preyed on poultry (*n* = 265). Overall, sheep accounted for the highest proportion of livestock losses, representing 30.65% (*n* = 648) of all reported predator kills (Table 2). A statistically significant difference was observed in the mean number of livestock types lost to different predators (*F*_6,35_ = 2.39, *p* < 0.05).

**TABLE 2:** Livestock losses due to different predators in the adjacent villages of JWPF from 2019 to 2021.

Livestock	Predators						Total	Mean
	Hyena	Baboon	Leopard	Raptors	Wildcat	Jackal		
Donkey	179	0	0	0	0	0	179	13.17
Sheep	162	257	167	19	26	17	648	108
Goat	55	140	156	4	5	0	360	60
Horse	47	0	0	0	0	0	47	7.83
Mule	37	0	0	0	0	0	37	6.17
Cattle	298	0	17	0	0	0	315	52.5
Poultry	0	159	0	265	84	20	528	88
Total	778	556	340	288	115	37	2114	

**FIGURE 2:**
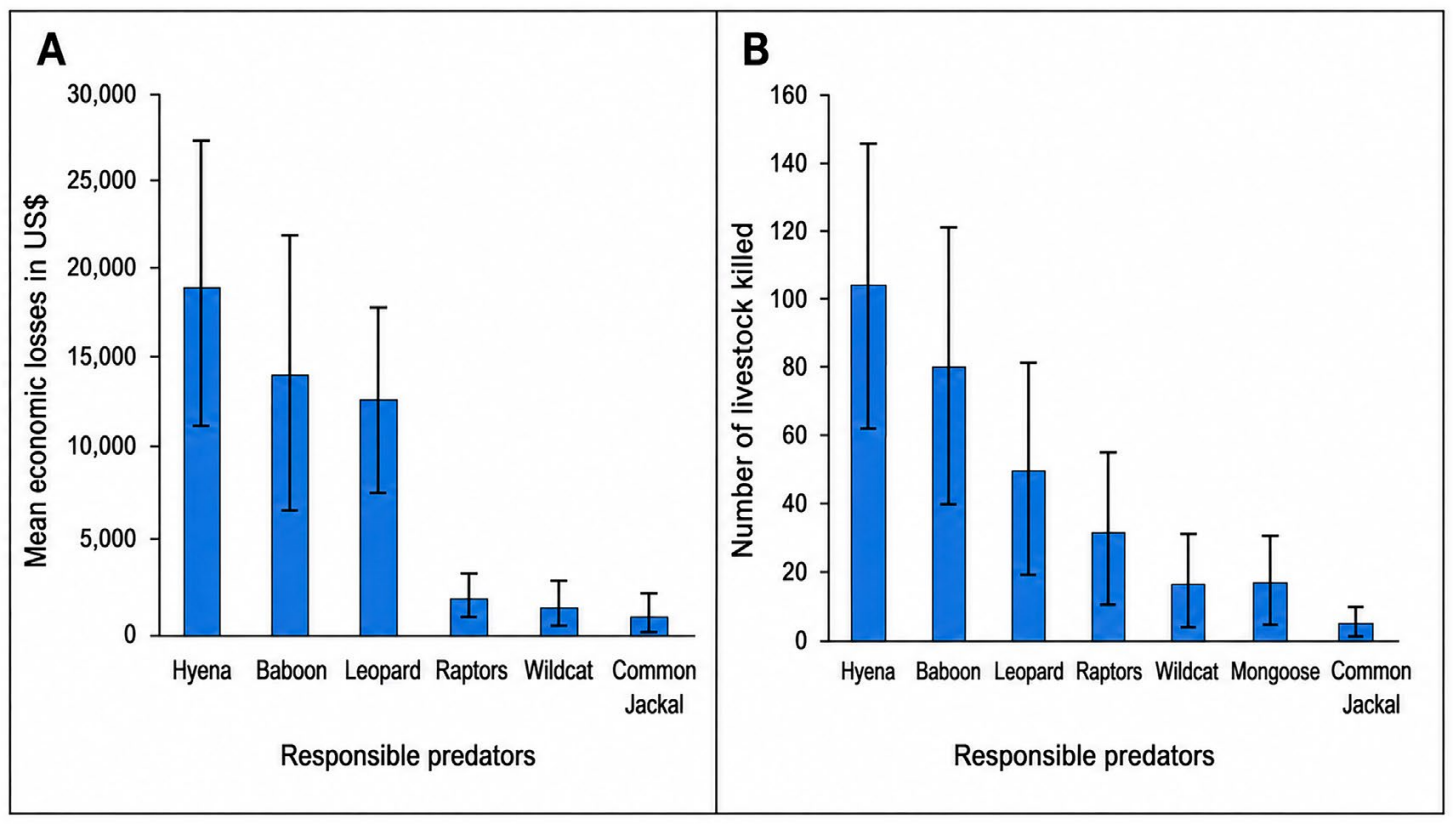
Livestock losses (Mean ± SE) (B) and corresponding economic costs (Mean ± SE) (A) attributed to predators around JWPF from 2019 to 2021.

Respondents reported that six carnivore species caused 2114 livestock losses over 2‐year period. Of these, spotted hyenas accounted for 778 (36.80%) of the losses, followed by olive baboons, which killed 556 head of livestock (26.30%). Other predators, including leopards 340 (16.08%), raptors 288 (13.62%), and African wildcats 115 (5.44%), were also reported to have caused livestock losses in the area. The common jackal accounted for only 37 livestock losses (1.75%) in the study area (Figure 2B). On average, spotted hyenas killed 103.57 (SE = ±39.21), olive baboons killed 79.43 (SE = ±39.87), and leopards killed 48.57 (SE = ±29.28) livestock during 2019–2021. The mean number of livestock killed by different predator species varied significantly (*F*_6,35_ = 3.57, *p* < 0.05).

### 3.3 Livestock Depredation and Economic Loss due to Carnivores

**FIGURE 3:**
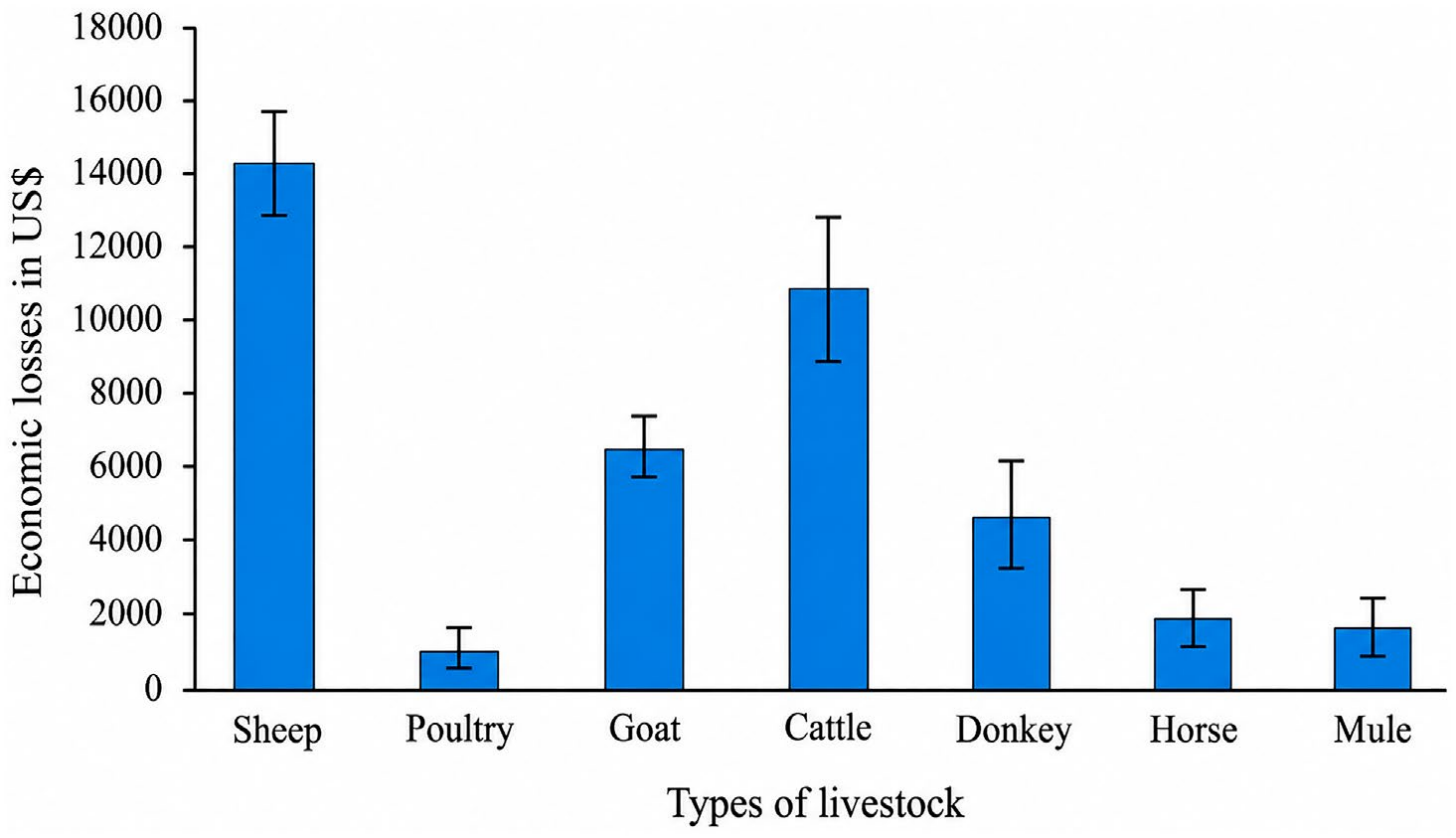
Total economic losses (Mean ± SE) of each livestock killed by predators around JWPF from 2019 to 2021.

A total of 2114 livestock losses to predators resulted in an estimated economic loss of $207,810. Of all predators, spotted hyenas were responsible for the highest mean economic losses at $19,263 (SE ± 7248), followed by olive baboons at $14,153 (SE ± 7741), leopards at $12,818 (SE ± 4959), and raptors at $1700 (SE ± 689) (Figure 2A). However, African wildcats and common jackals accounted for the lowest mean economic losses, at about $1383 ± 747 and $1035 ± 835, respectively. There was a significant difference in the mean economic losses attributed to predator species (*χ* ^2^ (5) = 11.97, *p* = 0.035). Of the total economic losses ($20,490) attributed to livestock species, sheep accounted for 34.78% (mean $14,256 ± 560.5), followed by cattle at 26.55% ($10,836 ± 1475.6), and goats at 16.07% ($6480 ± 617.7). However, the lowest economic losses were recorded for horses at 4.52% ($1880 ± 512.3), mules at 4.52% ($1776 ± 344.5), and poultry at 2.55% ($1056 ± 206.3) (Figure 3).

Spotted hyenas, olive baboons, and leopards were identified as the top three predators responsible for most livestock losses in the study area (Appendix S2). All donkeys (179, 100%), horses (47, 100%), and mules (37, 100%) killed were attributed to spotted hyenas. However, spotted hyenas, olive baboons, and leopards were responsible for 25%, 39.66%, and 25.77% of sheep losses, respectively. Similarly, the three predators (hyenas, olive baboons, and leopards) were responsible for 15.28%, 38.89%, and 43.33% of goat losses, respectively, in the study area. In addition to sheep and goats, olive baboons were responsible for 30.11% of poultry losses. The average number of sheep killed by the top three predator species varied significantly (*F*_2,18_ = 6.023, *p* < 0.05). Sheep, poultry, goats, and cattle were the top four livestock species killed by predators around the JWPF. Sheep accounted for the largest share of recorded kills, 648 (30.65%), followed by poultry, 528 (24.98%), goats, 360 (17.03%), and cattle, 315 (14.90%) (Appendix S3).

### 3.4 Livestock Killed by Predators From 2019 to 2021

A total of 192 (100%) respondents confirmed livestock depredation by predators in the study area. They reported a total loss of 2114 livestock around JWPF between 2019 and 2021, with sheep accounting for the highest losses (*n* = 648), followed by poultry (*n* = 528) and goats (*n* = 360). The fewest losses were recorded for horses (*n* = 47) and mules (*n* = 37). Livestock depredation varied among villages surrounding JWPF, with the highest numbers of kills reported in Siba Silassie (*n* = 558) and Siba Dalo (*n* = 447), while the lowest was recorded in Asgori Sora (*n* = 314). Similarly, the highest economic loss was reported in Siba Silassie (49,426 $), whereas the least loss was recorded in Asgori Sora (27,180 $) (Table 3).

**TABLE 3:** Number of livestock killed and its estimated economic losses in each village around JWPF from 2019 to 2021.

Livestock	Villages				
	Arbu Abba Gada	Siba Silassie	Siba Dalo	Asgori Sora	Siba Kobbi
Sheep	125 (13,750)	139 (15,290)	144 (15,840)	117 (12,870)	123 (13,530)
Poultry	53 (530)	175 (1750)	84 (840)	93 (930)	123 (1230)
Goat	77 (6930)	93 (8370)	75 (6750)	53 (4770)	62 (5580)
Cattle	64 (11,008)	78 (13,416)	74 (12,728)	30 (5160)	69 (11,868)
Donkey	31 (4030)	60 (7800)	51 (6630)	13 (1690)	24 (3120)
Horse	18 (3600)	8 (1600)	12 (2400)	4 (800)	5 (1000)
Mule	12 (2880)	5 (1200)	7 (1680)	4 (960)	9 (2160)
Total kill/Economic loss ($)	380 (42,728)	558 (49,426)	447 (46,868)	314 (27,180)	415 (38,488)

**FIGURE 4:**
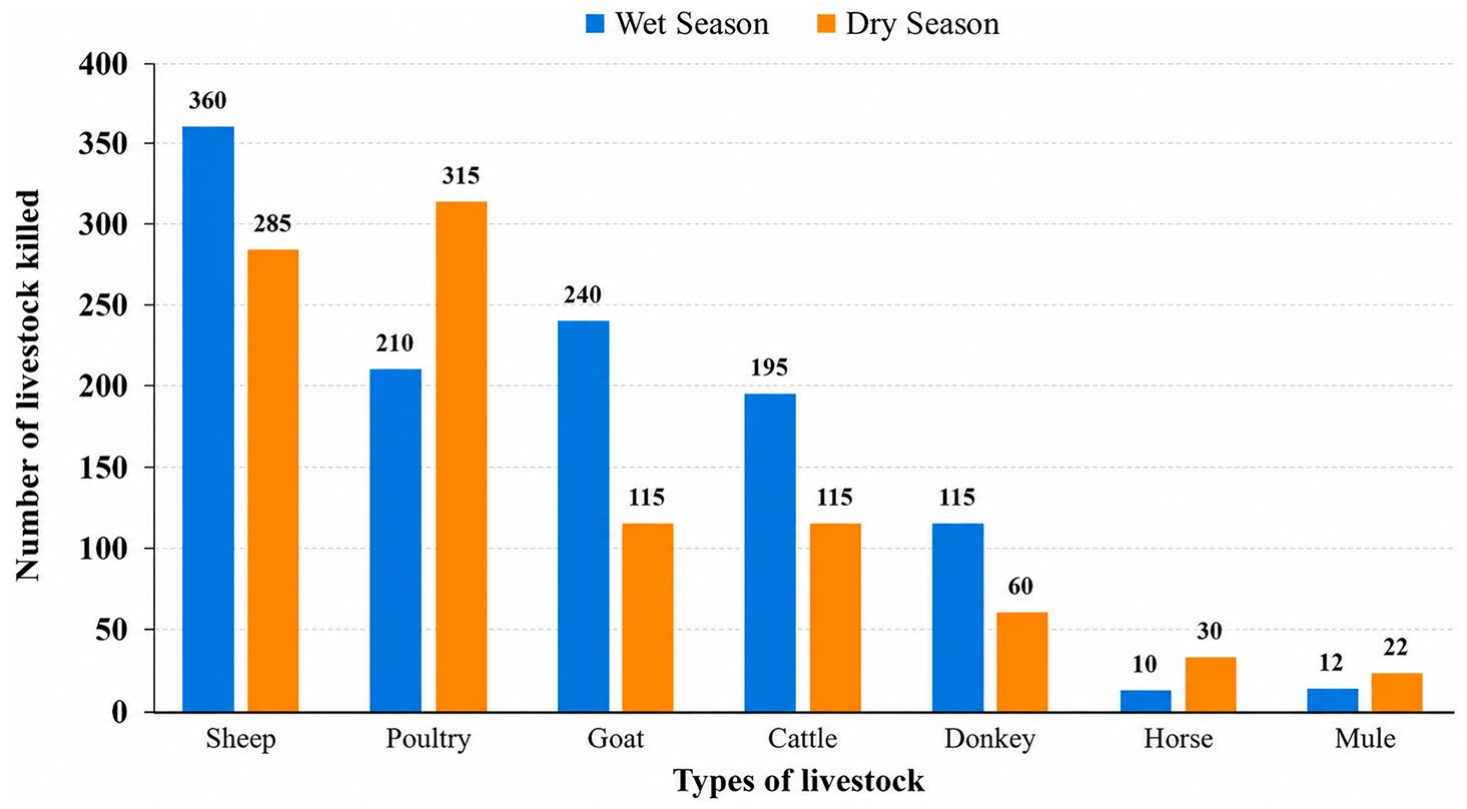
Number of livestock killed during the wet and dry seasons around JWPF from 2019 to 2021.

Seasonal livestock depredation by carnivorous animals is shown in Figure 4. Respondents reported that livestock losses were generally higher in the wet season than in the dry season. Of 2114 domestic animals killed in the study area, 1157 (54.73%) and 957 (45.27%) were lost during the wet and dry seasons, respectively. The difference in livestock depredation between the two seasons was statistically significant (*χ* ^2^ = 18.92, df = 1, *p* < 0.05). Moreover, seasonal variation in the number of sheep, goats, poultry, cattle, donkeys, and horses killed was significant (*χ* ^2^ = 8.45, df = 1, *p* < 0.05; *χ* ^2^ = 22.05, df = 1, *p* < 0.05; *χ* ^2^ = 10.24, df = 1, *p* < 0.05; *χ* ^2^ = 10.41, df = 1, *p* < 0.05; *χ* ^2^ = 9.08, df = 1, *p* < 0.05; *χ* ^2^ = 5.63, df = 1, *p* < 0.05, respectively), whereas the number of mules killed did not differ significantly between seasons (*χ* ^2^ = 3.27, df = 1, *p* > 0.05).

### 3.5 Mitigation Measures Used for Human‐Carnivore Conflict

**FIGURE 5:**
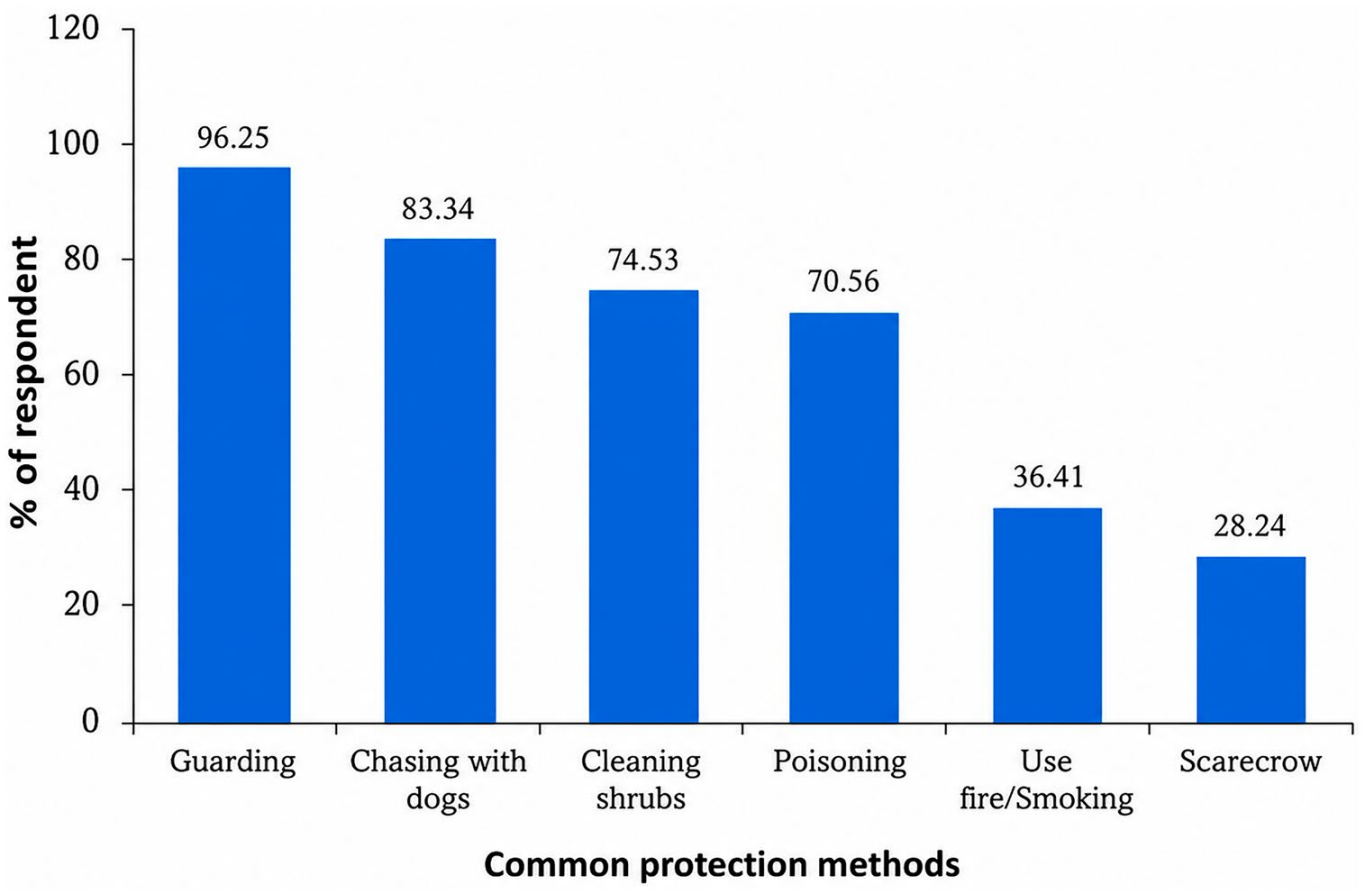
Mitigation measures used against crop damage and livestock depredation around JWPF.

In the study area, farmers employed a range of techniques to protect their livestock from predators. Mitigation strategies included guarding (watching), night watch, clearing or removing shrubs around the home, keeping livestock in the barn, using fire or a torch, and using scarecrows. Most respondents (96.25%) reported that guarding is an effective control measure against livestock depredation, followed by chasing with dogs (83.34%) and clearing shrubs (74.53%) around the homes. The least‐used mitigation method was scarecrows (28.24%) in the study area (Figure 5). The use of these mitigation techniques varied significantly among farmers in the study area (*χ* ^2^ = 55.33, df = 5, *p* < 0.05).

## 4 Discussion

### 4.1 Causes and Types of Human‐Wildlife Conflict

Human–wildlife conflicts occur worldwide and are driven by multiple factors (Messmer 2000; Thirgood et al. 2005; Madden 2008; Lamarque et al. 2009). These include livestock depredation and crop damage, the absence of buffer zones, uncontrolled livestock grazing in the forest, and shifting cultivation adjacent to the protected area. In this study, livestock depredation and crop damage were identified as significant drivers of human‐carnivore conflict. The observed conflicts were attributed to local communities encroaching on protected areas, agricultural expansion, and unregulated livestock grazing. A similar study by Biset et al. (2019) reported that both crop and livestock damages were associated with human‐wildlife conflict in and around Borena Sayint National Park, northern Ethiopia. Previous studies have also shown that households located close to protected areas are more susceptible to livestock depredation (Linkie et al. 2007; Karanth et al. 2013), a pattern linked to inadequate buffer zones (Muboko et al. 2016). Insufficient or absent buffer zones can intensify livestock depredation and crop damage around protected areas (Biset et al. 2019).

In the present study, all respondents whose farmlands were categorized as near reported severe incidences of crop and livestock depredation. In contrast, about 84.21% and 51.72% of respondents whose farmlands were categorized as medium and far, respectively, reported incidences of human–wildlife conflict. This result indicates that HWC intensifies as people get closer to protected areas (Kidane et al. 2024). Similar studies conducted in various parts of Ethiopia and other African countries have also revealed that the frequency of human‐wildlife conflict increases near protected areas (Hariohay and Røskaft 2015; Alelign and Yonas 2017; Biset et al. 2019; Horgan and Kudavidanage 2020; Pisa and Katsande 2021). Human encroachment and the expansion of agricultural activities around protected areas result in the overexploitation of natural resources and the reduction of core habitat areas used by large wild animals. Since large animals travel longer distances to fulfill their daily needs, reduced core habitat areas would increase overlaps in resource and habitat use between humans and large mammals, as well as between large mammals and livestock. As reported by Messmer (2000), HWC arises when the requirements of wildlife overlap with human needs, which can intensify conflict. In particular, large mammals may be affected by retaliatory killings around protected areas (Dahal et al. 2022). Such ongoing retaliatory killings could lead to future declines in large mammal populations in the area. Similar outcomes have been documented in the Wabe Valley of Bale, Ethiopia (Atickem et al. 2010).

### 4.2 Livestock Lost to Different Predators

Predation on livestock by predators represented another form of human–wildlife conflict around the JWPF. Eight predator species, including raptors, were reported to cause livestock losses around the JWPF. Olive baboons, leopards, and spotted hyenas were primarily responsible for livestock losses in the study area. Similarly, Alemu et al. (2017) reported that baboons, spotted hyenas, and leopards caused the highest losses of various domestic animals each year in and around the Gemshat forest area, Wollo, Ethiopia. The olive baboon has been recognized as the most troublesome species, acting as both a predator of livestock and a crop raider around the JWPF. In the present study area, olive baboons have been known to hunt lambs, goats, cattle, and poultry. Similarly, olive baboons were reported as the top problematic animal in Banja Woreda, Awi Zone, Ethiopia (Derebe et al. 2022). The olive baboon is a dietary and habitat generalist mammal widely reported for depredation and crop raiding in various parts of the world. Several studies have documented olive baboons as crop raiders and livestock predators (Anthony and Wasambo 2009; Derebe et al. 2022). The incidence of livestock depredation by spotted hyenas and leopards was reported in and around Chebera‐Churchura National Park (Demeke and Afework 2013; Acha et al. 2018) and the Gemshat forest area, Wollo, Ethiopia (Alemu et al. 2017). Moreover, both spotted hyenas and leopards were reported as principal predators responsible for livestock depredation in Bale Mountains National Park (Atickem et al. 2010). Contrary to this, the prevalence of livestock depredation was very low around Midre‐Kebid Abo Monastery, Southwest Ethiopia (Temesgen et al. 2022), which could be due to effective conflict mitigation measures, distance from the forest, and lower predator density in the area. Moreover, keeping livestock in permanent enclosures near the home can reduce livestock depredation.

### 4.3 Livestock Losses and Its Economic Estimates

Six predators were responsible for a total of 2114 livestock losses around JWPF during 2019 and 2021. Most respondents reported occasional increases in cattle depredation during this period. The high rate of cattle depredation in the study area may be attributed to changes in prey behavior, habitat encroachment, and inadequate husbandry practices (Dickman 2008). As reported by Ladan (2014), human‐wildlife conflicts are intense in many African countries due to unrestrained encroachment into wildlife habitats. Furthermore, inadequate night enclosure construction is linked to significant cattle losses around protected areas (Odden et al. 2014). Spotted hyenas, olive baboons, and leopards were reported to be responsible for the highest livestock losses in the study area. Moreover, hyenas were reported to have caused the highest losses of cattle and all other livestock except poultry around JWPF. Similarly, studies have shown that spotted hyenas prey on all types of livestock in the Sariska Tiger Reserve, Rajasthan, India (Sekhar 1998), and in Lake Nakuru National Park and Soysambu Conservancy, Kenya (Koskey et al. 2021). Among the killed livestock, all donkeys, horses, and mules were killed exclusively by hyenas. Similarly, Temesgen et al. (2022) reported that hyenas were responsible for the loss of all cattle, donkeys, horses, and mules in the surrounding areas of Alage College, the Central Rift Valley of Ethiopia. This could be because hyenas move in clans, which helps them manage large livestock more easily. Hyenas have also been implicated in the highest rates of depredation on cattle, sheep, goats, and donkeys in the Dhera‐Dilfaqar Block of Arsi Mountains National Park, southeastern Ethiopia (Tufa et al. 2018).

Leopards around the Jorgo‐Wato Protected Forest (JWPF) primarily preyed on sheep, goats, and young cattle because these smaller animals are easier for predators to target (Bibi et al. 2013; Ahmad et al. 2016). Small ruminants are generally more vulnerable to carnivores than large ungulates because they are easier to kill and transport to safe feeding sites (Dar et al. 2009; Ahmad et al. 2016). Livestock depredation by large carnivores is often more frequent in areas with intensive human activity and livestock husbandry (Sijapati et al. 2021). In addition, habitat shrinkage, forest degradation, and fragmentation can intensify human‐carnivore conflict by forcing predators into closer contact with people and domestic animals (Mills et al. 2023). Similar findings from the buffer zone of Banke National Park in Nepal identified leopards as the major livestock predator, with conflicts linked to declining natural prey, water scarcity, and poor livestock guarding practices (Subedi et al. 2020; Kabir et al. 2014). Comparable conditions may exist around JWPF, where bushmeat hunting for subsistence and commercial purposes likely reduces wild prey populations (Erena et al. 2020). Furthermore, livestock grazing inside the forest during the dry season, when surrounding grazing areas become depleted, may expose domestic animals to greater predation risk. Therefore, conserving wild prey species and improving livestock protection measures are important for reducing human‐leopard conflict in the area. In contrast, the common jackal caused comparatively lower livestock losses around JWPF.

During the present study, livestock depredation caused total economic losses of 207,810 US$ over 2 years, representing a substantial burden on communities that depend heavily on livestock for their livelihoods. Comparable losses have been reported globally, underscoring the widespread socioeconomic impacts of human–carnivore conflict around protected areas. For example, livestock depredation in the buffer zone of the Jigme Khesar Strict Nature Reserve in Bhutan resulted in losses of 25,722.51 US$, while annual losses of 48,490 US$ were reported in northwestern Pakistan (Wangchuk et al. 2018; Khattak et al. 2021). In Africa, livestock losses attributed to carnivores such as lions reached approximately 130,000 US$ in western and central regions (Bauer et al. 2001), whereas depredation by wild dogs in northern Kenya caused losses of 2870 US$ within 3 months (Woodroffe et al. 2005). Similarly, sheep and lamb depredation in the USA resulted in direct losses of 16.5 million US$ (Jones 2004). These findings demonstrate that livestock depredation significantly affects the livelihoods of rural households worldwide, highlighting the importance of improved livestock husbandry practices to reduce economic losses (Gidey and Bauer 2010). In the present study, hyenas accounted for the highest economic losses (55.62%, 115,576 US$), followed by olive baboons (20.43%, 42,460 US$) and leopards (18.50%, 38,454 US$). Similar patterns were reported in Kenya, where hyenas were identified as the leading predators causing severe livestock losses (Muriuki et al. 2017; Koskey et al. 2021).

### 4.4 Seasonal Variation in Human‐Carnivore Conflict

Livestock depredation shows seasonal patterns (Holmern et al. 2007). According to this study, most livestock attacks increase during the rainy season rather than the dry season. Similar patterns were observed near Tsavo National Park in Kenya (Patterson et al. 2004) and Waza National Park in Cameroon (Bauer 2003). During the wet season, livestock limit their movement around homesteads because they can easily find food nearby. However, this may increase the dispersal of predators from the forest into human residential areas. Consequently, heavy rain, fog, and taller shrubs and annual plants around homesteads and adjacent grazing lands may reduce livestock vigilance. These conditions can provide cover for predators, enabling them to ambush and capture livestock during the dry season. In contrast, during the dry season, when annual herbs are dried and the ground is cleared and brighter, livestock and herders have improved visibility and vigilance, reducing the risk of predation.

### 4.5 Mitigation Measures Used for Human‐Carnivore Conflict

Livestock depredation poses a major threat to household food security for communities living near the Jorgo‐Wato Protected Forest (JWPF), making effective local conflict mitigation strategies essential for both livelihoods and biodiversity conservation. The choice of mitigation measures often depends on effectiveness, cost, conflict type, and local suitability (Muruthi 2005). Communities around JWPF employ several traditional methods to reduce livestock losses, including guarding livestock, chasing predators with dogs, clearing shrubs and herbs around homes and farmlands, poisoning carcasses, using fire or smoke near livestock enclosures, and scaring wildlife with noise. Among these, guarding livestock during both daytime grazing and nighttime enclosure was identified as the most common and effective strategy. Similar findings have been reported from other protected areas in Ethiopia and elsewhere in Africa, where guarding remains a widely practiced indigenous approach to reducing crop and livestock damage (Mwakatobe et al. 2013; Mekonen 2020; Shanko et al. 2021; Sime et al. 2022; Yazezew 2022; Gehring et al. 2010). Guarding is relatively inexpensive, environmentally sustainable, and effective in minimizing human–wildlife conflict (Lamarque et al. 2009). However, it often requires continuous human presence, and in many households children are assigned guarding responsibilities, which can contribute to school absenteeism (Girmay and Teshome 2015). Additionally, the absence of a buffer zone around JWPF has forced many farmers to abandon farmland near the forest because of persistent wildlife damage, resulting in significant socioeconomic and grazing land losses.

## 5 Conclusion and Recommendations

This study found that human–carnivore conflict is a significant livelihood and conservation challenge for communities around the JWPF. Livestock depredation was the primary form of conflict, causing substantial economic losses for local people. The spotted hyena, olive baboon, and leopard accounted for the largest share of livestock and monetary losses among the six carnivore species involved. The study also showed that local people use indigenous conflict mitigation measures, such as guarding animals, scaring predators with domestic dogs, and clearing vegetation around homes to reduce the risk of attacks. This study provides the first baseline information on the prevalence, severity, and economic impact of human–carnivore conflict around JWPF. The study underscores the value of integrated management approaches that balance increased livestock security measures, population sensitization, and conservation planning. Strengthening community‐led conflict mitigation efforts and promoting greater local participation in wildlife management will be crucial to ensuring human‐large mammal coexistence around JWPF. Moreover, a framework for compensating property losses and providing alternative forms of employment or income for local people shall be designed to minimize HWC in the area.

## Author Contributions

**Mosissa Geleta Erena:** conceptualization (lead), data curation (equal), formal analysis (lead), funding acquisition (equal), investigation (lead), methodology (lead), project administration (equal), resources (equal), software (equal), supervision (equal), validation (equal), visualization (equal), writing – original draft (lead), writing – review and editing (lead). **Diriba Diba:** conceptualization (equal), data curation (equal), funding acquisition (equal), investigation (equal), project administration (equal), resources (equal), validation (equal), visualization (equal), writing – review and editing (equal).

## Funding

This work was supported by Wollega University, Thematic Research Grant.

## Ethics Statement

The research was approved by the Wollega University, Department of Biology, and conformed to Ethiopian regulatory standards.

## Conflicts of Interest

The authors declare no conflicts of interest.

## Supporting information


**Appendix S1:** Demographic characteristics of respondents in the study area (*N* = number of respondents).
**Appendix S2:** Percentages of different livestock killed by the top three predators around JWPF.
**Appendix S3:** Top four livestock types killed by predators around JWPF from 2019 to 2021.

**Data S1:** ece374156‐sup‐0002‐supinfo.docx.

## Data Availability

The data that support the findings of this study are available from Dryad Digital Repository at https://doi.org/10.5061/dryad.q83bk3jrp.
