# Supplementary material for: Human‐Carnivore Conflicts and Associated Economic Losses in Villages Adjacent to Jorgo‐Wato Protected Forest, Western Ethiopia
Source: Ecol Evol. 2026 Aug 9;16(8):e74156. doi: 10.1002/ece3.74156 (PMC13454211; doi:10.1002/ece3.74156)
Supplement: Supplementary file 1 [file ECE3-16-e74156-s001.docx]

**Appendices**

Appendix 1. Demographic characteristic of respondents in the study area (N = number of respondents)

| **Category** | | **N** | **%** |
| --- | --- | --- | --- |
| **Gender** | Male | 147 | 76.56 |
|  | Female | 45 | 23.44 |
| **Marital status** | Married | 142 | 73.95 |
|  | Unmarried | 38 | 19.79 |
|  | Others | 12 | 6.25 |
| **Educational status** | Illiterate | 93 | 48.44 |
|  | <grade 6 | 47 | 24.48 |
|  | Grade 7-10 | 34 | 17.71 |
|  | Grade 10-12 | 18 | 9.38 |
| **Livelihood activities** | Crop cultivation | 124 | 64.58 |
|  | Livestock rearing | 108 | 56.25 |
|  | Mixed farming | 156 | 81.25 |
| **Average distance of respondent’s farmland** | Far | 29 | 15.10 |
|  | Medium | 95 | 49.48 |
|  | Near | 68 | 35.42 |
| **HWC reported by residents of varying distances** | Far | 15 | 51.72 |
|  | Medium | 80 | 84.21 |
|  | Near | 68 | 100 |
| **Causes of conflicts** | Livestock depredation | 95 | 49.48 |
|  | Crop damage | 64 | 33.33 |
|  | Absence of buffer zone | 18 | 9.38 |
|  | Livestock grazing in the forest | 12 | 6.25 |
|  | Shifting cultivation | 3 | 1.56 |
|  | Others | 0 | 0 |

Appendix 2. Percentages of different livestock killed by the top three predators around JWPF

% of livestock killed by top 3 predators

| Types of livestock | Hyena | Baboon | Leopard |
| --- | --- | --- | --- |
| Donkey | 100 | 0 | 0 |
| Sheep | 25 | 39.66 | 25.77 |
| Goat | 15.28 | 38.89 | 43.33 |
| Horse | 100 | 0 | 0 |
| Mule | 100 | 0 | 0 |
| Cow | 94.6 | 0 | 0 |
| Poultry | 0 | 30.11 | 0 |

Appendix 3. Top four livestock types killed by predators in around JWPF from 2019-2021

| Types of livestock | Numumber of livestock killed |
| --- | --- |
| Sheep | 648 |
| Poultry | 528 |
| Goat | 360 |
| Cattle | 315 |
