# Supplementary material for: Human‐Carnivore Conflicts and Associated Economic Losses in Villages Adjacent to Jorgo‐Wato Protected Forest, Western Ethiopia
Source: Ecol Evol. 2026 Aug 9;16(8):e74156. doi: 10.1002/ece3.74156 (PMC13454211; doi:10.1002/ece3.74156)
Supplement: Supplementary file 2 [file ECE3-16-e74156-s002.docx]

**Wollega University**

**Department of Biology**

**Questionaries prepared for interview to collect data on Human-carnivore conflicts and associated economic losses in villages adjacent to Jorgo-Wato Protected Forest, Western Ethiopia**

Dear respondents,

The purpose of the study is to collect factual and pertinent data regarding conflicts between humans and carnivores in the vicinity of JWPF. Information obtained from you will assist the wildlife conservation authorities in striking a balance between the interests of the local community and the large wild mammals in the area. Hence, we kindly ask you to provide genuine response for all question presented to you in the form of interviews.

Thank you for your response!

**Date**: ____________________________________

**Name of interviewer**: _______________________

**Name of village**: ___________________________________

**QUESTIONARY PRESENTED TO RESPONDENTS IN THE FORM OF INTERVIEW**

**1. Demographic characteristics**

A. **Gender**: Male ______	Female________
B. **Age class**: 15-20	21-30	31-40	41-50	51-60	above 60
C. **Marital status:** Married	Unmarried	Others
D. **Educational level**: Illiterate	<grade 6	Grade 7-10	Grade 10-12
E. **Livelihood activities**: Crop cultivation	Livestock rearing	Mixed farming
F. **Distance of respondent from forest:** **Far** ≥5km	**Medium** (3-4km)	**Near** ( 1-2km)
G. **HWC reported by residents of varying distances**:	Far	Medium	near

**2. Human wildlife related questionaries**

A. How much is the distance of your cultivation land from forest edge?

A. 0-2km (near) B.2.1-4km (medium) C. 4.1-5km (far)

B. What are the causes of conflicts between human-carnivore in the area?
I. Livestock depredation
II. Absence of buffer zone
III. Livestock grazing in the forest
IV. Shifting cultivation
V. Others
C. Lists the name of common predators responsible for conflicts around JWPF

| No | Name of carnivores | Stated reason for the conflicts |
| --- | --- | --- |
| 1 |  |  |
| 2 |  |  |
| 3 |  |  |
| 4 |  |  |
| 5 |  |  |
| 6 |  |  |
| 7 |  |  |

3. How many domestic animals were lost due to human-carnivore conflicts in the past two year?

| **No** | **Predator** | **livestock lost** | **Number lost** |
| --- | --- | --- | --- |
| 1 |  |  |  |
| 2 |  |  |  |
| 3 |  |  |  |
| 4 |  |  |  |
| 5 |  |  |  |
| 6 |  |  |  |

4. During which season of a year does more livestock were lost in the area**?**

A. Dry season	B. Wet season

5. Write during which season of a year does the following livestock lost to predators?

| **No** | **Loss of livestock** | **Season** | |
| --- | --- | --- | --- |
|  |  | **Wet** | **Dry** |
| 1 | Sheep |  |  |
| 2 | Poultry |  |  |
| 3 | Goats |  |  |
| 4 | Cattle |  |  |
| 5 | Donkey |  |  |
| 6 | Horse |  |  |
| 7 | Mule |  |  |

6. What were the ranks of carnivore species most involved in livestock depredation? List the most problematic to the least.

| 1 |  |
| --- | --- |
| 2 |  |
| 3 |  |
| 4 |  |
| 5 |  |
| 6 |  |

9. What are the carnivore species most involved in depredation of livestock?

| 1 |  |
| --- | --- |
| 2 |  |
| 3 |  |
| 4 |  |
| 5 |  |
| 6 |  |

10. What are the top ranked problematic carnivores that cause conflicts in the area?

| 1 |  | 5 |  |
| --- | --- | --- | --- |
| 2 |  | 6 |  |
| 3 |  | 7 |  |
| 4 |  | 8 |  |

11. What control or preventative measures do the local people use against livestock depredation in the study area?

| 1 |  | 5 |  |
| --- | --- | --- | --- |
| 2 |  | 6 |  |
| 3 |  | 7 |  |
| 4 |  | 8 |  |

**Questionnaire for key informants and focus group discussions on Human -large Mammals conflicts**

1. What are the main causes of human-carnivore conflicts around Jorgo Wato Protected Forest?
2. Which carnivore species were commonly depredating livestock around Jorgo Wato Protected Forest?
3. What types of livestock’s were most attacked by carnivores around Jorgo Wato Protected Forest?
4. How do you prevent carnivore species from attacking livestock?
5. What mitigation majors do you recommend to minimize human-carnivore conflicts around Jorgo Wato Protected Forest?
6. Do you think that human-carnivore conflicts have an impact on the economy of the local community?
